# Plant Growth Promotion Under Water: Decrease of Waterlogging-Induced ACC and Ethylene Levels by ACC Deaminase-Producing Bacteria

**DOI:** 10.3389/fmicb.2018.01096

**Published:** 2018-05-25

**Authors:** Sajid Ali, Won-Chan Kim

**Affiliations:** School of Applied Biosciences, College of Agriculture and Life Sciences, Kyungpook National University, Daegu, South Korea

**Keywords:** Bacterial ACC-deaminase, plant growth promotion, stress ethylene, flooding, terrestrial and riparian plants

## Abstract

Some plant growth-promoting bacteria encode for 1-aminocyclopropane-1-carboxylate (ACC) deaminase, which facilitates plant growth and development by lowering the level of stress ethylene under waterlogged conditions. The substrate ACC is the immediate precursor for ethylene synthesis in plants; while bacterial ACC deaminase hydrolyzes this compound into α-ketobutyrate and ammonia to mitigate the adverse effects of the stress caused by ethylene exposure. Here, the structure and function of ACC deaminase, ethylene biosynthesis and waterlogging response, waterlogging and its consequences, role of bacterial ACC deaminase under waterlogged conditions, and effect of this enzyme on terrestrial and riparian plants are discussed.

## Introduction

Quantitative methods, such as those used to produce climate change models, suggest that the frequency and severity of heavy precipitation may increase in the near future all over the worldwide (Milly et al., 2002; Wright et al., 2017). Natural vegetation and economically important crops are negatively affected by waterlogging. The development of plants with high yield under waterlogged conditions is one of the primary objectives of breeding crops for sustainable agriculture (Osakabe et al., 2014; Pedersen et al., 2017). Under waterlogged conditions, plant roots typically become hypoxic and accumulate free oxygen, which leads to abiotic stress conditions (Glick, 2014). Plants have evolved complex physiological and biochemical adaptations to fine-tune their responses to a variety of environmental stresses (Ravanbakhsh et al., 2017). In response to waterlogging stress, in addition to temporal, physiological, and biochemical changes, plants produce different enzymes and stress proteins to mitigate the adverse effects of stress (Li et al., 2012; Osakabe et al., 2014).

Several microorganisms interact with plants during their lifespan. These microorganisms may be beneficial, neutral, or harmful to plants. Particularly, plant–bacterial interactions manifest in different modes; these interactions may be symbiotic (bacteria live inside the root nodules), endophytic (bacteria live inside the plant tissues), rhizospheric (bacteria bind to the root surface), or phylloshperic (bacteria bind to the leaf or stem surface) (Glick, 1995; Hallmann et al., 1997; Kozdrój and Van Elsas, 2000; Knief et al., 2012). The bacteria, which occur in soil and are beneficial to plants, are commonly known as plant growth-promoting bacteria (PGPB; Glick et al., 2007). These PGPB use different strategies to promote plant development and stress mitigation. Numerous studies have demonstrated the positive effects of different microorganisms growing near stressed plants (Glick et al., 2007; Li et al., 2012; Glick, 2014; Kang et al., 2014; Nascimento et al., 2014). In this regard, bacteria that produce the enzyme ACC deaminase are very important. ACC deaminase cleaves the substrate ACC into ammonia and α-ketobutyrate (Honma and Shimomura, 1978). A high concentration of ethylene can lead to growth inhibition, chlorosis, or even death of plants. Thus, bacterial ACC deaminase likely plays a pivotal role in decreasing the excessive amount of ethylene by catabolizing its precursor (ACC) into ammonia and α-ketobutyrate and alleviating its effects which are induced by waterlogging stress (Glick et al., 2007; Sasidharan et al., 2017). In this review, the response of plants to waterlogged conditions, potential role of ACC deaminase-producing bacteria, and effects on land and riparian plants are discussed.

## Plant anaerobiosis and bacterial ACC deaminase

**Figure 1:**
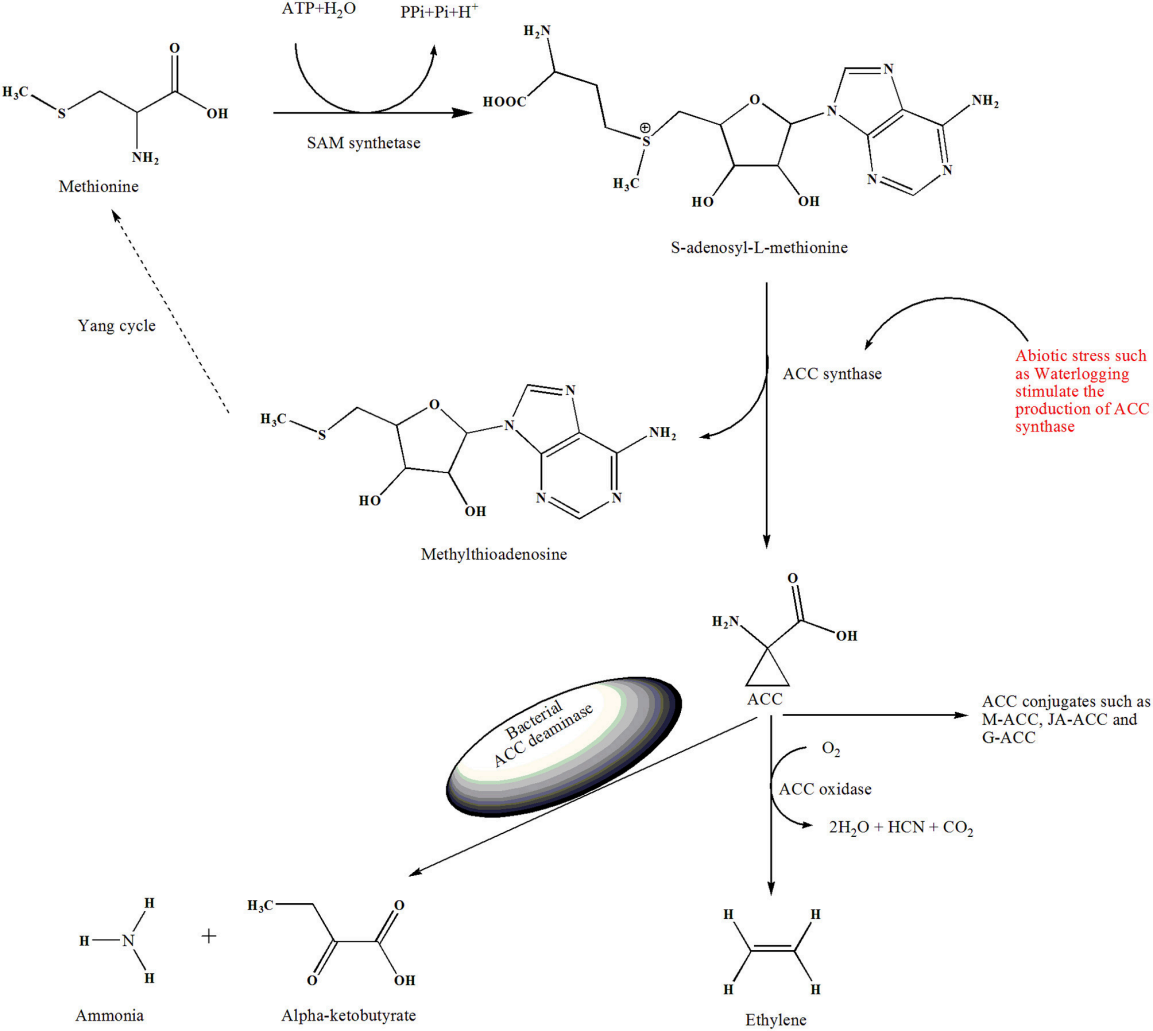
Demonstrate the overview of ethylene biosynthesis and role of ACC deaminase-producing bacteria in the stress condiction. The enzyme SAM-synthetase (SAMS) convert amino acid methionine to *S*-adenosyl-L-methionine (SAM). Then ACC synthase (ACS) convert SAM into ACC, in this reaction 5′methylthioadenosine (MTA) is also produced, which is recycled back to methionine, this multi-step pathway is also known as the Yang cycle. ACC oxidase convert ACC to ethylene in the presence of oxygen. Besides, ACC can be conjugated to M-ACC, JA-ACC, G-ACC by the action of their respective enzymes. Plant response to waterlogged condition and produce excess amount of ACC. ACC deaminase–producing bacteria catabolize the exuded ACC into ammonia and alpha-ketobutyrate and mitigate the adverse effects of waterlogging stress on the plant.

Anaerobiosis can be defined as “life in the absence of free oxygen,” which is a potential threat to aerobic organisms (Pedersen et al., 2017). Plants require molecular oxygen to maintain their normal functions. Hypoxic conditions can adversely affect the developmental stages and even the survival of plants (Paul et al., 2016). The ultimate response of a plant to waterlogging stress is the up-regulation of certain genes and production of stress-related biomolecules (Voesenek and Sasidharan, 2013). Under hypoxic conditions, plant roots produce increased amounts of ACC synthase, which converts *S*-adenosyl-L-methionine (SAM) to 1-aminocyclopropane-1-carboxylic acid (ACC) in greater amounts (Glick, 2014; Sasidharan et al., 2017). Upon oxidation, ACC is converted to ethylene by ACC oxidase (Figure 1). ACC oxidase requires molecular oxygen to cause conformational changes and split ACC to produce ethylene, which is the gaseous hormone of plants. Under waterlogging stress, increased amounts of ACC cannot be oxidized and converted to ethylene because of the unavailability of molecular oxygen (Glick et al., 2007). Thus, ACC is transported to the shoot and is converted to ethylene by ACC oxidase. In this manner, waterlogged plants produce increased amounts of ethylene (20-fold higher than in the non-submerged tissue within 1 h), leading to physiological and anatomical damages (Sasidharan et al., 2017). Chlorosis, necrosis, and low productivity are the most prominent symptoms produced in plants exposed to prolonged waterlogged conditions (Mayak et al., 2004; Glick et al., 2007; Paul et al., 2016). The PGPB that produce ACC deaminase typically reduce the ACC and ethylene levels by approximately 2- to 4-fold and mitigate the adverse effects of waterlogging stress on the plants (Grichko and Glick, 2001a; Mayak et al., 2004).

### Waterlogging and its consequences

Natural and human induced waterlogged conditions are equally responsible for the loss of cash crops, delayed cultivation operations, and low yield. The condition in which soil is fully saturated with water and an anaerobic environment prevails is known as waterlogging (Setter and Waters, 2003; Nishiuchi et al., 2012). Under waterlogged conditions, the soil profile is completely saturated with water and the water table rises to a level in which the soil pores in the crop root zone are saturated and resulting in the restriction of the normal supply of oxygen. While submerged, the major portion or all of the plant is covered with water and facing unfavorable conditions such as low light intensity, restricted gas diffusion, and effusion of soil nutrients. The diffusion of oxygen in water is ~10,000-fold slower than in air, and when the soil is waterlogged, the flux of oxygen is ~320,000-fold lower because of water fills the soil pores (Colmer and Flowers, 2008; Nishiuchi et al., 2012). The main causes of waterlogging include poor soil quality, heavy rainfall, inadequate drainage system, poor irrigation management, and natural drainage obstruction. Similarly, variable weather patterns and land locked parches with no outlets can also lead to either temporary or permanent waterlogged conditions. In 2007, FAO reported that ~30 million hectares of irrigated land area were affected by waterlogging which resulted into low crop yield in different regions of the world (Setter and Waters, 2003; Najeeb et al., 2015).

Under waterlogged condition, the plant roots become hypoxic or oxygen-limited, leading to harmful effects on plant growth and development. In response to hypoxic conditions, plants produce increased amounts of ACC synthase to convert SAM into ACC, which is the ultimate substrate for ethylene synthesis (Li et al., 2012; Glick, 2014). Additional ACC formed in the roots of waterlogged plant is transported to the shoots for conversion into ethylene in the presence of oxygen (Jackson, 1985; Glick, 2014). The accumulation of stress caused by ethylene in plant tissues increases the rate of reactive oxygen species generation, leading to the destruction of macromolecules, inhibition of photochemical performance, and ultimately causing cell death (Grichko and Glick, 2001a; Glick, 2005; Ahmed et al., 2006). According to De Klerk and Hanecakova (2008), ethylene has a dual role, as it triggers or inhibits the rooting of mung bean cuttings, which depends on the stage of the rooting process. They revealed the role of ethylene during rooting and concluded that additional ethylene has opposite effects in the successive phases of rooting. Initially, the effect of ethylene is promotive, but later becomes inhibitory and depends on the auxin concentration and immersion level (De Klerk and Hanecakova, 2008).

Recently, plant adaptation to water stresses in the form of submergence or waterlogging has been extensively studied in different plant species. There are a numerous of strategies adopted by the plants under water stress, although the rate of acclimatization varies by species and with the threshold of stress. In excess water stress, plants experience a deficient oxygen supply and undergo several physiological, morphological, and metabolic changes to survive in submerged or waterlogged environments (Najeeb et al., 2015). In this regard, rapid shoot elongation during submergence, development of aerenchyma (in grasses), increased intercellular spaces in roots, and development of adventitious roots are the alternative strategies induced under waterlogged conditions by different plants species (Bailey-Serres and Voesenek, 2008; Vidoz et al., 2010; Najeeb et al., 2015). However, the deleterious effects of deficient oxygen in a waterlogged environment causes accumulation of by-products of fermentation (ethanol, acetaldehyde) in roots and toxic compounds (phenolic acid, hydrogen sulfide) in soil, causing chlorosis, necrosis, and ultimately plant death (Zeng et al., 2013; Bailey-Serres and Colmer, 2014; Glick, 2014; Najeeb et al., 2015).

In some of plant roots, ethylene plays role in acclimating to waterlogged condition by promoting lysigenous aerenchyma formation and interacting with auxin to initiate adventitious root formation (He et al., 1994). Further studies are required to confirm the spatial and temporal response of plants to ethylene under waterlogged conditions. Under waterlogging stress, the higher concentration of ethylene produced may be responsible for the stressful condition and cause epinasty, inhibited nodulation in legumes, reduced chlorophyll content, increased aging rate, and senescence and leaf abscission promotion, inhibited root elongation, and low growth and development (Singh et al., 2015). One method for mitigating this stress is through ACC deaminase-producing bacteria, which lower the excessive amounts of ACC exuded from plant roots (Glick, 2014).

### Ethylene biosynthesis and waterlogging stress

Ethylene is a gaseous phytohormone with a very simple two-carbon structure, which is formed through the breakdown of the amino acid methionine. Despite its simple structure, ethylene is involved in many aspects of the plant life cycle, including seed germination, root nodulation, flower senescence, fruit ripening, and leaf abscission (Johnson and Ecker, 1998; Wang et al., 2002; Vanderstraeten and Van Der Straeten, 2017). The role of methionine as a biological precursor of plant hormone ethylene was first reported by Lieberman et al. (1966), while the biochemistry of ethylene biosynthesis has been extensively reported by numerous researchers (Lieberman et al., 1966; Hoffman et al., 1982; Wang et al., 2002; Vanderstraeten and Van Der Straeten, 2017).

Ethylene biosynthesis in plants is highly regulated by different developmental and environmental factors. Normally, in plants, methionine is converted to SAM through the action of SAM synthetase. However, ACC synthase converts SAM into ACC and yields 5′-methyl-thio-adenosine (MTA) in the same reaction (Yang and Hoffman, 1984). The 5′-methyl-thio-adenosine produced in this reaction is converted into methionine in a different cycle known as the Yang cycle (Yang and Hoffman, 1984; Bleecker and Kende, 2000; Vanderstraeten and Van Der Straeten, 2017). In the Yang cycle, ethylene can be synthesized continuously, as the methyl group is recycled to produce ethylene (Figure 1). The synthesis of ACC from SAM has also been reported in microbial species such as *Penicillium citrinum* (Jia et al., 1999; Wang et al., 2002). The genes encoding of ACC synthase and ACC oxidase exist as a multigene family, and induced expression of ACC oxidase results in conversion of ACC to ethylene (John, 1997). In this final step, CO_2_ and cyanide are also generated; cyanide is detoxified by β-cyanoalanine synthase (Wang et al., 2002; Vanderstraeten and Van Der Straeten, 2017; Nascimento et al., 2018).

Generally, two different strategies are used by plants to survive under waterlogged conditions: quiescence (a type of dormancy or temporary termination of the activities to conserve the internal energy) and escape (an amelioration response to stimulate growth under waterlogged conditions, and also known as low oxygen escape syndrome) (Bailey-Serres and Voesenek, 2008). Ethylene plays a vital role in regulating of these strategies (Sasidharan and Voesenek, 2015). Normally, ethylene is found inside plant tissues at very low concentrations (0.01 μL/L) and regulates the growth and development of plants (Reid, 1995). Various physical and biological factors affect and modulate the amount of ethylene produced in a specific plant (Abeles et al., 2012), which leads to the production of ethylene stress in response to the stressful conditions (Glick et al., 2007). Glick et al. (1998) presented a model for ethylene stress, which revealed that generation of ethylene peaks twice; the first ethylene peak is only a small fraction of the second peak. The first small peak reflects the consumption of ACC (ethylene precursor) present prior to the stressed condition whereas the second, larger peak arises after the synthesis of additional ACC in response to the stressed condition (Glick et al., 1998).

Under waterlogged conditions, plant roots suffer from hypoxia and express a high amount of ACC compared to well-aerated roots (Drew, 1997; Morgan and Drew, 1997). Under hypoxic conditions, plants overexpress ACC synthase genes, resulting in enhanced ACC synthase activity in roots within 3–12 h after the initiation of waterlogging (Shiono et al., 2008). Previous studies of the members of the ACS gene family showed that these genes are differentially regulated at the transcriptional level in a tissue- or organ-specific manner in different plant species (De Paepe and Van Der Straeten, 2005; Vanderstraeten and Van Der Straeten, 2017). In tomato plants, the ACS7, ACS3, and ACS2 genes are successively expressed in roots under hypoxic conditions (Grichko and Glick, 2001b). Peng et al. (2005) proposed two different signaling pathways in *Arabidopsis* for the synthesis of ACC synthase under hypoxic conditions. Their results showed that four (*ACS*2, *ACS*6, *ACS*7, and *ACS*9) of the 12 *ACS* genes in *Arabidopsis* were induced during waterlogging. They also revealed that one pathway leads to activation of *ACS*2, *ACS*6, and *ACS*7, whereas the other pathway leads to the activation of *ACS*9. However, the expression of these genes was highly regulated by the concentration of ethylene present in the plant tissues (Peng et al., 2005). Genes such as ACS and ACO regulate not only the biosynthesis of ethylene, but also regulate the formation of ACC derivatives which greatly contribute to the ethylene concentration in plants. Typically in plants, all synthesized ACC is not only converted to ethylene, but also some may be converted into other conjugates such as *N*-malonyl-ACC, ⋎-glutamyl-ACC, and jasmonyl-ACC through the action of ACC-malonyltransferase, g-glutamyl-transpeptidase, and Jasmonic acid resistance 1, respectively (Hoffman et al., 1982; Martin et al., 1995; Staswick and Tiryaki, 2004; Nascimento et al., 2018). Furthermore, it has been reported that these conjugates can be reconverted into ACC and transported into other parts of the plant such as the shoot through the phloem, where it catabolized into ethylene in the presence of oxygen (Hoffman et al., 1982; Morris and Larcombe, 1995; Wang et al., 2002).

Similarly, studies of the role of ethylene as a stress hormone provided a foundation for understanding ACC transport in plants. Several researchers have reported the mechanism and also demonstrated the short (ACC compartmentalization in the vacuole) and long distance (root to shoot) transport of ACC and its conjugates in different plants species (Vanderstraeten and Van Der Straeten, 2017; Nascimento et al., 2018). Under waterlogged conditions, the low amount of oxygen directs the expression of ACS genes and a greater amount of ACC is formed in the roots. The rate of conversion of ACC into ethylene is suppressed because of the unavailability of molecular oxygen, which is required for the oxidation and conversion of ACC into ethylene. As ACC is transported into the shoot, the expression of ACO genes is promoted and a high amount of ethylene is produced (Glick, 2014; Vanderstraeten and Van Der Straeten, 2017). In waterlogged tomato plant roots, the increased amount of ACC is formed and transported through the xylem to the shoots where it is rapidly converted into ethylene and causes leaf epinasty and abscission (Bradford and Yang, 1980; Grichko and Glick, 2001b; Wessjohann et al., 2003). Similarly, Peng et al. (2005) and Geisler-Lee et al. (2009) investigated the differential expression of the ACS and ACO genes in response to waterlogged conditions in *Arabidopsis* and maize plants, respectively. ACC is released from the plants roots into the rhizosphere, which is absorbed by ACC deaminase-producing bacteria such as *Pseudomonas* and utilized as a carbon and nitrogen source (Glick et al., 1998; Penrose et al., 2001).

Li et al. (2013) used a proteomic approach to identify the PGPB response and proteins in cucumber roots under hypoxic conditions (Table 1). They revealed that *P. putida* UW4 and hypoxic stress stimulate gene expression in cucumber roots and investigated the regulation of protein and metabolic pathways. This was a pragmatic approach for the identification of enzymes involved in the interaction between plants and PGPB in a waterlogged environment (Li et al., 2013).

**Table 1:** List of studies on applications ACC deaminase-producing plant growth promoting bacteria under waterlogged conditions.

**Plant name**	**Bacteria used**	**Experimental design**	**Waterlogging stress**	**Evaluated characteristics: physiological, biochemical, or molecular**	**Objectives results conclusion**	**References**
*Lycopersicon esculentum*	*Pseudomonas* sp. UW4	Growth chamber Tomato seeds in petri dishes, incubated with 5 mL bacterial suspension for 1 h to allow bacteria to bind to seed coat. Five days prior to onset of waterlogging, 240 mL bacterial suspension with an absorbance of 0.5 at 600 nm were added.	Waterlogging stress applied on 55 days old tomato plants for 9 consecutive days	ACC deaminase activity Ethylene measurement Epinasty measurement Chlorophyll Contents Shoot fresh weight Shoot dry weight	**Objectives:** To investigate the effect of ACC deaminase producing bacteria on plants under waterlogging condition. **Results:** Plants grown from bacterized seeds showed a significant tolerance to waterlogging stress. **Conclusion:** Bacteria may act as a sink for ACC, thereby ameliorating some of the damage to plants caused by waterlogged condition.	Grichko and Glick, 2001b. *Plant Physiology and Biochemistry*.
*Cucumis sativus* L. cv.	*Pseudomonas* sp. UW4	Sterilized seeds treated with *P. putida* UW4 and control seeds were planted in sterilized calcinated clay in growth pots in greenhouse. At 2-leaf stage, seedlings were selected and transferred into pots with 1/2 Hoagland's nutrient solution. After 8 days growth observed. Proteins were identified by LTQ-MS/MS.	72 h of hypoxic (test) or non-hypoxic (control) treatment with and without PGPB	Identification and functional classification of proteins in cucumber seedling roots, PGPB effect on biomass, Antioxidants, Survey of Defense stress proteins	**Objectives:** To investigate plant growth-promoting bacteria responsive proteins and metabolism in cucumber roots under hypoxic stress through proteomic survey. **Results:** Protein spots detected from cucumber roots in the absence of stress:1980, presence of *P. putida* UW4:1893, Hypoxic condition and PGPB: 1735 **Conclusion:** *P. putida* UW4 significantly released the inhibition of hypoxic stresses on plants biomass. The protein profiles of the roots of cucumber seedlings in response to the *P. putida* UW4 and hypoxia suggested how the protein and metabolism pathways are regulated.	Li et al., 2013. *Journal of Proteomics*
*Brassica napus*	*Pseudomonas* sp.UW4	*In situ* (field site) Bacterial suspension of OD600 = 0.5 (~3 × 10^8^ CFU mL^−1^). The bacterial suspension placed in sterile plastic screw cap 500 mL bottles and stored on ice for immediate transport to the field plot. Next, 2 mL of the bacterial suspension was applied per seed hole and the surrounding area (4 cm diameter).	High waterlogging stress (less than 50% plant survival) Intermediate waterlogging stress (~50% plant survival) Low waterlogging stress (More than 50% plant survival) And elevated soil Ni concentration	Canola germination and Survival. Effect of ACC deaminase producing PGPB Root biomass Shoot biomass Shoot length	**Objectives:** To investigate transgenic and non-transgenic canola plants in the presence of ACC deaminase-producing bacteria under waterlogging and metal stress conditions. **Results:** Under waterlogged conditions, ACC deaminase-producing transgenic canola and canola treated with *P. putida* UW4 Showed greater shoot biomass. Under high waterlogging stress the shoot biomass was reduced and Ni accumulation was increased in all instances relative to under low waterlogged conditions. **Conclusion:** Transgenic canola and *P. putida* UW4 increased plant biomass, separately or in combination, under stress conditions.	Farwell et al. (2007). *Environmental Pollution*.
*Ocimum sanctum*	*Achromobacter xylosoxidans*, *Serratia ureilytica*, *Herbaspirillum seropedicae* *Ochrobactrum rhizosphaerae*	Growth chamber *Ocimum*, 30 days old seedlings were transplanted to soil pots of 10 inches diameter in a glass house using autoclaved soil. Each pot contained one seedling treated with isolated bacterial inoculum 10^8^ CFU mL^−1^.	Waterlogging applied at the level of 2 cm above the soil surface for 15 days. The pots under waterlogging conditions without bacterial inoculation served as positive control and pots without waterlogging condition and without bacterial culture acted as negative control.	ACC deaminase activity Ethylene production, Chlorophyll concentration, Lipid peroxidation, Proline concentration	**Objectives:** To investigate the usefulness of ACC deaminase-containing bacteria and its effect in ameliorating the damage of waterlogged condition. **Results:** Plants showed high yield 46.5% higher than uninoculated stressed plants. **Conclusion:** ACC deaminase-producing bacteria greatly contribute to protecting plants from waterlogging stress.	Barnawal et al. (2012). *Plant physiology and biochemistry*.
*Cicer arietinum*	*Mesorhizobium ciceri* strainLMS-1	Growth chamber Bacterial suspension ODs were adjusted so that there were ~10^9^ CFU mL^−1^ Four replicates were used per treatment; Plants were harvested 31, 38, and 45 days after inoculation.	Waterlogging stress in container filled with 1 cm above the soil surface. Waterlogging was applied for 7 days (21 days after inoculation).	*Mesorhizobium ciceri* strain transformation with the *acdS* gene of *Pseudomonas putida* UW4. Plasmid used pRKACC. Plant growth promotion ability of the LMS-1 (pRKACC) transformed strain under normal and waterlogging conditions were verified. ACC deaminase and nitrogenase activity were also measured.	**Objectives:** To evaluate the nodulation performance of *Mesorhizobium* strain transformed with an exogenous ACC deaminase gene and plant growth under normal and waterlogged conditions. **Results:** Transformed strain LMS-1 showed 127% increased ability to nodulate chickpea and 125% promotion of the growth of chickpea compared to the wild-type strain, under normal conditions. LMS-1 wild-type strain showed a higher nodule number under stress than under control conditions. **Conclusion:** The use of rhizobial strains with improved ACC deaminase activity can play a pivotal role in developing microbial inocula for agricultural purposes.	Nascimento et al. (2012). *Plant and soil*.
*Rumex palustris*	*Pseudomonas putida* WT (Wild type which encodes for ACC deaminase and Mutant lacking ACC deaminase)	Growth chamber Bacterial suspension with an absorbance of 0.5 at 600 nm were used. Plants inoculation with one of three bacterial treatments Uninoculated, control, WT, mutant. Plants with four leaves were selected based on homogeneity of developmental stage.	Waterlogging stress in container: Complete submergence, three days for short-term and 17 days submergence for long-term.	Effect of inoculum producing ACC deaminase on short and long-term submergence responses on RIPARIAN PLANT. Root-associated bacterial population densities were measured. The plant height, fresh and dry weight were also measured.	**Objectives:** To investigate the effect of ACC deaminase-producing bacteria and not producing bacteria on waterlogged tolerant riparian plant. To study short and long-term submergence and modulating plant responses on *Rumex palustris*. **Results:** There is a negative effect of ACC deaminase on riparian plants. As total shoot fresh weight and leaf size increased significantly after 72 h submergence from 1.06 to 2.13 g and leaf length increased from 7.25 to 66.22 mm after 72 h by ACC deaminase-deficient mutant. While ACC deaminase-producing bacteria (WT) reduce total fresh weight from 3.01 to 2.56 g and also decreased elongation of the youngest leaf (*F* = 6.32; *P* = 0.007). **Conclusion:** Root-associated bacteria can alter plant response to environmental stress by altering plant hormonal balance. Plant-microbe interactions may be reckoned in assessing of plant ecological adaptations.	Ravanbakhsh et al. (2017). *Journal of Ecology*.

### Role of bacterial ACC deaminase under waterlogged conditions

Pioneering studies of the stress ethylene model suggested that bacterial ACC deaminase is induced by increasing amounts of its substrate, ACC (Glick et al., 1998). The increased amount of ACC is produced by ACC synthase inside the plant tissues after exposure to waterlogging stress, which directly induces the production of ACC deaminase inside bacteria (Glick, 2014). The regulation and differential expression of *acdS* depends on the availability of oxygen, and concentrations of ACC and ethylene. The DNA sequence of the ACC deaminase gene suggests that this segment contains several features involved in the transcriptional regulation of this gene. Mainly, *acdR* (ACC deaminase regulatory gene) is present in the upstream region of *acdS* gene and encodes for leucine-responsive regulatory protein, which regulates the transcription of *acdS* (Vanderstraeten and Van Der Straeten, 2017; Nascimento et al., 2018). Under hypoxic conditions, transcription from the *acdS* promoter regions may be modulated by leucine-responsive regulatory protein and fumarate nitrate reduction regulatory protein (Duan et al., 2013; Glick, 2014). A detailed model of the transcriptional regulation of *acdS* was developed by Glick et al. (2007) and Li et al. (2013). Under hypoxic conditions, the plant response to stress and an increased amount of ACC is produced in the roots. A significant amount of ACC is exuded from plant roots, which is hydrolyzed to ammonia and α-ketobutyrate by ACC deaminase-producing bacteria. In this manner, the amount of ACC is get decreased inside plant tissue and the equilibrium is maintained through exudation of additional ACC into the rhizosphere. The availability of the substrate ACC to rhizospheric bacteria enhances the expression of *acdS* and an increases the amount of ACC deaminase (Glick et al., 1998, 2007; Li and Glick, 2001; Nascimento et al., 2018). Similarly, transgenic plants expressing *acdS* may be constructed by using different promoters; a promoter that is root-specific and anaerobically inducible such as *rolD* would appear to be ideal under hypoxic conditions (Grichko and Glick, 2001b).

The enzymes ACC oxidase and ACC deaminase compete for their common substrate. The content of ethylene in plants under waterlogged conditions is reduced if ACC deaminase reacts before the induction of ACC oxidase (Glick, 2014). Depending on the concentration gradient, ACC that is released is cleaved to form α-ketobutyrate and ammonia. Using this strategy, symbiotic bacteria lower the levels of ethylene stress (by 60–90%) by producing ACC deaminase and contribute to the growth and development of plants under waterlogged conditions (Penrose et al., 2001; Mayak et al., 2004; Saleem et al., 2007).

## Bacterial ACC-deaminase

### Discovery and occurrence

Honma and Shimomura (1978) initially isolated ACC deaminase from bacterium *Pseudomonas* sp. ACP and yeast *Hansenula saturnus*. They also suggested that this enzyme requires pyridoxal phosphate for its activity. The molecular weight and *Km* value of the enzyme varies with the source. The molecular weight of ACC deaminase isolated from *Pseudomonas* sp. ACP is 104,000 Da and its *Km* is 1.5 mM, whereas the molecular weight of ACC deaminase isolated from *H. saturnus* (recently known as *Cyberlindnera saturnus*) is 69,000 Da and it has a higher *Km* value of ~2.6 mM (Honma and Shimomura, 1978; Nascimento et al., 2014). This enzyme has also been reported in *Pseudomonas chloroaphis* 6G5, *Pseudomonas putida* GR12-2, and *P. putida* UW4 (Klee et al., 1991; Jacobson et al., 1994; Hontzeas et al., 2004; Glick, 2005). Recently, several studies investigated the production of ACC deaminase from endophytic bacteria and fungi (Viterbo et al., 2010; Rashid et al., 2012; Xu et al., 2014; Khan et al., 2016; Sarkar et al., 2017).

DNA sequence analysis revealed the presence of putative ACC deaminase genes in the plant genomes (Glick, 2005). The structural gene *acdS* is responsible for producing ACC deaminase and has been reported in different organisms. The proposed model for ACC deaminase evolution and phylogenetic analysis showed that the origin of ACC deaminase in bacteria dates to *Actinobacteria*. *Meiothermus* is a representative of *Deinococcus thermus*, which is one of the earliest bacterial lineages and contains *acdS* in its chromosome. A study by Nascimento et al. (2014) enhanced our understanding of the location of *acdS* in the genome of bacterial species and showed that *acdS* is vertically inherited in most bacterial species (Nascimento et al., 2014).

### Structure and function

The molecular mass of a monomeric subunit of ACC deaminases is ~36,500–42,000 Da (Honma, 1985; Glick, 2005; Glick et al., 2007). The enzyme uses one molecule of pyridoxal 5-phosphate per subunit as an essential cofactor, which is required for its normal function. ACC deaminase acquires a compact structure by folding into two domains, each of which has an open twisted alpha/beta structure, and one molecule of pyridoxal 5-phosphate is completely concealed inside the enzyme (Yao et al., 2000; Nascimento et al., 2014). The crystalline structures of *Pseudomonas* sp. ACP and *Hansenula saturnus* ACC deaminase have been determined, which demonstrates the important amino acid residues for substrate recognition and catalysis. Similarly, the alignment of amino acid residues demonstrate that the active sites of ACC deaminase of *H. saturnus* and *Pseudomonas* sp. are identical and conserved (Yao et al., 2000; Glick et al., 2007).

For appropriate functioning, the optimal temperature required by ACC deaminase ranges from 26 to 30°C and the optimum pH is ~8.0–8.5 (Yao et al., 2000; Glick, 2014). Previous studies confirmed that ACC deaminase is not secreted but rather contained within the cytoplasm of bacteria. Under waterlogged conditions, excessive ACC is produced inside the plant roots, which is released from the plant tissues. Exuded ACC is absorbed and cleaved by ACC deaminase-producing bacteria attached to the plant roots (Jacobson et al., 1994; Glick, 2005).

### Ecological significance

In symbiotic relationship, plants and bacteria interact and communicate with each other. The plant provides a carbon source to the associated bacteria, and in return bacteria provide access to the nutrients present in the soil and possible protection from abiotic stress conditions. Based on the available information, in the rhizospheric ecological niche, soil is directly influenced by exudates from the plant roots and numerous different types of ACC deaminase producing bacteria and fungi may interact and communicate in response to different environmental stresses (Chien and Larsen, 2017). ACC deaminase-producing bacteria were mostly reported in the plant rhizosphere region and are nearly absent in the non-rhizospheric soil samples. Similarly, under waterlogged conditions, the increased amount of exuded ACC acts as a signaling molecule for ACC deaminase-producing bacteria in the rhizosphere.

The produced amount of ethylene inside plant tissues also influence plant-associated bacteria responses. These bacterial responses may be strain-specific and depend on the bacterial mode of action. For example, increased ethylene sensitivity in transgenic tomato plant caused a decline in the bacterial population (Ciardi and Klee, 2001; Nascimento et al., 2018). Similarly, the virulence gene expression of *Agrobacterium tumefaciens* is negatively affected by exogenous ethylene and leads to a decreased ability of T-DNA transfer and pathogenicity (Nonaka et al., 2008). Additionally, a study by Kim et al. (2007) demonstrated that the bacteria such as *P. fluorescens, P. aeruginosa* (PAO1), *P. putida*, and *P. syringae* can perceive and positively respond to ethylene produced by the plant (Kim et al., 2007). According to Augimeri and Strap (2015), ethylene causes overexpression of the cellulose synthesis operon of *Komagataeibacter xylinus* and contributes to the production of fruit-plants growth-promoting traits (Augimeri and Strap, 2015). Under waterlogged conditions, ACC and ethylene may act as signaling molecules, while ACC deaminase-producing bacteria modulate ethylene levels and mitigate the adverse effects on plant developmental stages. However, additional studies are needed to determine the role of some bacterial species in the utilization of ethylene as a carbon source and modulating plant growth. Moreover, ACC deaminase-producing bacteria have been used both in the laboratory and in the field to protect plants against growth inhibition caused by waterlogged conditions (Grichko and Glick, 2001b; Farwell et al., 2007).

Stress ethylene is produced under abiotic stress conditions, indicating that plants also produce higher amounts of ACC synthetase to produce a much higher level of ACC, which is released into the rhizosphere. In most cases, rhizospheric bacteria also encode enzymes for the production of indole acetic acid, which facilitates root exudation and enhances plant growth and development (Glick, 2014). The rhizospheric bacteria, which utilize ACC and produce indole acetic acid, display a reasonable advantage over other microorganisms (Glick et al., 1998; Glick, 2014).

A study by Timmusk et al. (2011) suggested that in the same rhizospheric ecological niche, more stressed ACC deaminase-producing *H. spontaneum* bacteria were present than in the rhizosphere of unstressed wild barley. This study revealed that ACC deaminase-producing bacteria survive freely in the rhizosphere of stressed host plants by utilizing ACC as a potential source of carbon and nitrogen and can directly contribute to decreasing stress ethylene in the plant (Timmusk et al., 2011; Nascimento et al., 2014).

## Mitigation of waterlogging induced stress by ACC deaminase-producing bacteria

### Effect of ACC deaminase-producing bacteria on land/terrestrial plants

ACC deaminase-producing bacteria can decrease the adverse effects of different environmental stresses on plants by increasing the symbiotic communication through signaling molecules (Grichko and Glick, 2001c). The bacteria, which produce ACC deaminase in association with plants under waterlogged conditions, lead to enhanced plant tolerance and can ameliorate the damage caused by the waterlogged condition (Table 1).

Importantly, an *in situ* study of Farwell et al. (2007) on *Brassica napus* suggested that *P. putida* UW4 produces ACC deaminase which ameliorates the damage of waterlogging and metal stress (Farwell et al., 2007). Following this reasoning, Barnawal et al. (2012) isolated rhizobacteria from the rhizosphere of waterlogged *Ocimum sanctum* on Dworkin and Foster salt minimal media by using the dilution plate technique and verified the ACC deaminase activity. The bacterial isolates were identified by 16S rDNA sequence analysis. The identified ACC deaminase-producing bacterial strains (*Achromobacter xylosoxidans, Serratia ureilytica, Herbaspirillum seropedicae*, and *Ochrobactrum rhizosphaerae*) were grown in 250-mL flasks for inoculation and the bacterial population was adjusted to 10^8^ CFU mL^−1^ in the broth medium prior to inoculation. Thirty-day-old seedlings of *O. sanctum* grown on autoclaved soil were exposed to waterlogging with water filled to a level of 2 cm above the soil surface for 15 consecutive days. Under waterlogged conditions, the maximum increase in fresh weight of seedlings was observed. The seedlings treated with ACC deaminase producing *Achromobacter xylosoxidans* (46.5%), followed by treatment with *Ochrobactrum rhizosphaerae* (45.1%), *Serratia ureilytica* (26.5%), and *Herbaspirillum seropedicae* (16.6%). They successfully demonstrated that like *O. sanctum*, other land plants were protected from waterlogging-induced damage by ACC deaminase-producing bacteria (Table 1; Barnawal et al., 2012). The higher concentration of ethylene and ACC inhibited nodulation numerous studies have revealed that the decreased amount of ethylene in leguminous plant roots enhances nodulation. A study by Nascimento et al. (2012) also suggested that the greater concentrations of ethylene and ACC inhibit roots nodulation, while the expression of *acdS* in LMS-1 (pRKACC) strain showed an increased nodule formation ability as well as increased total biomass of chickpea plants (Nascimento et al., 2012).

### Effect of ACC deaminase-producing bacteria on aquatic and riparian plants

ACC deaminase-producing bacteria have primarily been investigated for their ability to promote the growth of land plants under waterlogged conditions (Grichko and Glick, 2001b; Barnawal et al., 2012; Glick, 2014). However, Ravanbakhsh et al. (2017) demonstrated that ACC deaminase-producing bacteria have a negative effect on aquatic and riparian species. They used *Rumex palustris* plants in a controlled growth chamber, where plants were watered automatically to the level of saturation. The effect of ACC deaminase was evaluated by selecting two strains of *P. putida*, namely the wild type, which produced ACC deaminase, and an isogenic (mutant) type strain, which lacked the enzyme. A bacterial suspension (250 μL; OD600 = 0.5) was inoculated at the base of each plant. The results suggested that *R. palustris* produced a lower amount of ethylene in the presence of ACC deaminase-producing bacteria compared to the mutant strain (Table 1). The declining ethylene level limited the capabilities of *R. palustris* to express its normal morphological adaptation under waterlogged conditions (Ravanbakhsh et al., 2017).

Generally, ethylene and ACC act as inhibitors of nodulation initiated by rhizobial symbionts. However, in the physiology of aquatic and riparian plants, a higher concentration of ethylene plays a specific role, often as growth stimulator. Under hypoxic conditions *Sesbania rostrata* infected by *Azorhizobium caulinodans* required ethylene for rhizobial infection and nodule development. Under such conditions, the epidermis layer is bypassed by the rhizobia and ethylene appears to promote nodulation (D'Haeze et al., 2003; Guinel, 2015). Similarly, submergence of rice stimulates ethylene-mediated stem elongation and adventitious root formation (Lorbiecke and Sauter, 1999).

Thus, ACC deaminase can reduce the ethylene levels in plants required for signaling, adaptation, and survival of plants under waterlogged conditions. The growth and development of plants depend on their ecological perspectives; reducing in the ethylene levels in aquatic or riparian plants may lead to an antagonistic effect on plant adaptability, growth, and survival.

## Future prospects

This mini-review highlights that certain ACC deaminase-producing bacteria can help terrestrial plants tolerate the adverse effects of waterlogging and contribute to plant growth and development. ACC deaminase-producing bacteria mitigate stress ethylene and relieve the plants from these negative effects. Hence, it is important to determine the mechanism of plant root ACC exudation and bacterial amelioration of stress ethylene under waterlogged conditions. Studies of waterlogging effects in plant microbiome assembly will enhance our understanding of plant-microbe interactions in hypoxia and greatly contribute to successful agriculture in waterlogged regions. Because there is great variation in plant physiology and responses to waterlogged conditions, it is therefore, pivotally important to develop new strategies for more specific and efficient ACC deaminase-producing bacterial inoculants that can be used as alternatives to various agrochemicals in waterlogged areas.

Further studies are required to confirm the spatial and temporal responses of plants to ethylene under waterlogging conditions. Bacteria in the rhizosphere, which synthesize ACC deaminase, can alter plant responses to abiotic stress conditions by altering the hormonal balance in plants. Thus, plant–microbe interactions should be considered in the ecological context of the plant.

## Author contributions

All authors listed have made a substantial, direct and intellectual contribution to the work, and approved it for publication.

### Conflict of interest statement

The authors declare that the research was conducted in the absence of any commercial or financial relationships that could be construed as a potential conflict of interest.

## References

[B1] Abeles F. B. Morgan P. W. Saltveit M. E. Jr. (2012). Ethylene in Plant Biology. New York, NY: Academic Press.

[B2] Ahmed S. Nawata E. Sakuratani T. (2006). Changes of endogenous ABA and ACC, and their correlations to photosynthesis and water relations in mungbean (*Vigna radiata* (L.) Wilczak cv. KPS1) during waterlogging. Environ. Exp. Bot. 57, 278–284. 10.1016/j.envexpbot.2005.06.006

[B3] Augimeri R. V. Strap J. L. (2015). The phytohormone ethylene enhances cellulose production, regulates crp/fnrkx transcription and causes differential gene expression within the bacterial cellulose synthesis operon of Komagataeibacter (Gluconacetobacter) xylinus ATCC 53582. Front. Microbiol. 6:1459. 10.3389/fmicb.2015.01459 26733991 PMC4686702

[B4] Bailey-Serres J. Colmer T. D. (2014). Plant tolerance of flooding stress–recent advances. Plant Cell Environ. 37, 2211–2215. 10.1111/pce.12420 25074340

[B5] Bailey-Serres J. Voesenek L. A. (2008). Flooding stress: acclimations and genetic diversity. Annu. Rev. Plant Biol. 59, 313–339. 10.1146/annurev.arplant.59.032607.092752 18444902

[B6] Barnawal D. Bharti N. Maji D. Chanotiya C. S. Kalra A. (2012). 1-Aminocyclopropane-1-carboxylic acid (ACC) deaminase-containing rhizobacteria protect *Ocimum sanctum* plants during waterlogging stress via reduced ethylene generation. Plant Physiol. Biochem. 58, 227–235. 10.1016/j.plaphy.2012.07.008 22846334

[B7] Bleecker A. B. Kende H. (2000). Ethylene: a gaseous signal molecule in plants. Annu. Rev. Cell Dev. Biol. 16, 1–18. 10.1146/annurev.cellbio.16.1.1 11031228

[B8] Bradford K. J. Yang S. F. (1980). Xylem transport of 1-aminocyclopropane-1-carboxylic acid, an ethylene precursor, in waterlogged tomato plants. Plant Physiol. 65, 322–326. 10.1104/pp.65.2.322 16661182 PMC440319

[B9] Chien J. Larsen P. (2017). Predicting the plant root-associated ecological niche of 21 pseudomonas species using machine learning and metabolic modeling. arXiv. 1701.03220.

[B10] Ciardi J. Klee H. (2001). Regulation of ethylene-mediated responses at the level of the receptor. Ann. Bot. 88, 813–822. 10.1006/anbo.2001.1523

[B11] Colmer T. D. Flowers T. J. (2008). Flooding tolerance in halophytes. New Phytol. 179, 964–974. 10.1111/j.1469-8137.2008.02483.x 18482227

[B12] De Klerk G. J. Hanecakova J. (2008). Ethylene and rooting of mung bean cuttings. The role of auxin induced ethylene synthesis and phase-dependent effects. Plant Growth Regul. 56:203 10.1007/s10725-008-9301-8

[B13] De Paepe A. Van Der Straeten D. (2005). Ethylene biosynthesis and signaling: an overview. Vitam. Horm. 72, 399–430. 10.1016/S0083-6729(05)72011-2 16492477

[B14] D'Haeze W. De Rycke R. Mathis R. Goormachtig S. Pagnotta S. Verplancke C.. (2003). Reactive oxygen species and ethylene play a positive role in lateral root base nodulation of a semiaquatic legume. Proc. Natl. Acad. Sci. U.S.A. 100, 11789–11794. 10.1073/pnas.1333899100 12975522 PMC208836

[B15] Drew M. C. (1997). Oxygen deficiency and root metabolism: injury and acclimation under hypoxia and anoxia. Annu. Rev. Plant Biol. 48, 223–250. 10.1146/annurev.arplant.48.1.223 15012263

[B16] Duan J. Jiang W. Cheng Z. Heikkila J. J. Glick B. R. (2013). The complete genome sequence of the plant growth-promoting bacterium *Pseudomonas* sp. UW4. PLoS ONE 8:e58640. 10.1371/journal.pone.0058640 23516524 PMC3596284

[B17] Farwell A. J. Vesely S. Nero V. Rodriguez H. McCormack K. Shah S.. (2007). Tolerance of transgenic canola plants (*Brassica napus*) amended with plant growth-promoting bacteria to flooding stress at a metal-contaminated field site. Environ. Pollut. 147, 540–545. 10.1016/j.envpol.2006.10.014 17141927

[B18] Geisler-Lee J. Caldwell C. Gallie D. R. (2009). Expression of the ethylene biosynthetic machinery in maize roots is regulated in response to hypoxia. J. Exp. Bot. 61, 857–871. 10.1093/jxb/erp362 20008461 PMC2814119

[B19] Glick B. R. (1995). The enhancement of plant growth by free-living bacteria. Can. J. Microbiol. 41, 109–117. 10.1139/m95-015

[B20] Glick B. R. (2005). Modulation of plant ethylene levels by the bacterial enzyme ACC deaminase. FEMS Microbiol. Lett. 251, 1–7. 10.1016/j.femsle.2005.07.030 16099604

[B21] Glick B. R. (2014). Bacteria with ACC deaminase can promote plant growth and help to feed the world. Microbiol. Res. 169, 30–39. 10.1016/j.micres.2013.09.009 24095256

[B22] Glick B. R. Cheng Z. Czarny J. Duan J. (2007). Promotion of plant growth by ACC deaminase-producing soil bacteria. Eur. J. Plant Pathol. 119, 329–339. 10.1007/s10658-007-9162-4

[B23] Glick B. R. Penrose D. M. Li J. (1998). A model for the lowering of plant ethylene concentrations by plant growth-promoting bacteria. J. Theor. Biol. 190, 63–68. 10.1006/jtbi.1997.0532 9473391

[B24] Grichko V. P. Glick B. R. (2001a). Amelioration of flooding stress by ACC deaminase-containing plant growth-promoting bacteria. Plant Physiol. Biochem. 39, 11–17. 10.1016/S0981-9428(00)01212-2

[B25] Grichko V. P. Glick B. R. (2001b). Flooding tolerance of transgenic tomato plants expressing the bacterial enzyme ACC deaminase controlledby the 35S, rolD or PRB-1b promoter. Plant Physiol. Biochem. 39, 19–25. 10.1016/S0981-9428(00)01217-1

[B26] Grichko V. P. Glick B. R. (2001c). Ethylene and flooding stress in plants. Plant Physiol. Biochem. 39, 1–9. 10.1016/S0981-9428(00)01213-4

[B27] Guinel F. C. (2015). Ethylene, a hormone at the center-stage of nodulation. Front. Plant Sci. 6:1121. 10.3389/fpls.2015.01121 26834752 PMC4714629

[B28] Hallmann J. Quadt-Hallmann A. Mahaffee W. F. Kloepper J. W. (1997). Bacterial endophytes in agricultural crops. Can. J. Microbiol. 43, 895–914. 10.1139/m97-131

[B29] He C. J. Drew M. C. Morgan P. W. (1994). Induction of enzymes associated with lysigenous aerenchyma formation in roots of *Zea mays* during hypoxia or nitrogen starvation. Plant Physiol. 105, 861–865. 10.1104/pp.105.3.861 12232249 PMC160733

[B30] Hoffman N. E. Yang S. F. McKeon T. (1982). Identification of 1-(malonylamino) cyclopropane-1-carboxylic acid as a major conjugate of 1-aminocyclopropane-1-carboxylic acid, an ethylene precursor in higher plants. Biochem. Biophys. Res. Commun. 104, 765–770. 10.1016/0006-291X(82)90703-3 7073714

[B31] Honma M. (1985). Chemically reactive sulfhydryl groups of 1-aminocyclopropane-1-carboxylate deaminase. Agric. Biol. Chem. 49, 567–571.

[B32] Honma M. Shimomura T. (1978). Metabolism of 1-aminocyclopropane-1-carboxylic acid. Agric. Biol. Chem. 43, 1825–1831.

[B33] Hontzeas N. Zoidakis J. Glick B. R. Abu-Omar M. M. (2004). Expression and characterization of 1-aminocyclopropane-1-carboxylate deaminase from the rhizobacterium *Pseudomonas putida* UW4: a key enzyme in bacterial plant growth promotion. Biochim. Biophys. Acta 1703, 11–19. 10.1016/j.bbapap.2004.09.015 15588698

[B34] Jackson M. B. (1985). Ethylene and responses of plants to soil waterlogging and submergence. Annu. Rev. Plant Physiol. 36, 145–174. 10.1146/annurev.pp.36.060185.001045

[B35] Jacobson C. B. Pasternak J. J. Glick B. R. (1994). Partial purification and characterization of 1-aminocyclopropane-1-carboxylate deaminase from the plant growth promoting rhizobacterium Pseudomonas putida GR12-2. Can. J. Microbiol. 40, 1019–1025. 10.1139/m94-162

[B36] Jia Y. J. Kakuta Y. Sugawara M. Igarashi T. Oki N. Kisaki M.. (1999). Synthesis and degradation of 1-aminocyclopropane-1-carboxylic acid by *Penicillium citrinum*. Biosci. Biotechnol. Biochem. 63, 542–549. 10.1271/bbb.63.542 10227140

[B37] John P. (1997). Ethylene biosynthesis: the role of 1-aminocyclopropane-1-carboxylate (ACC) oxidase, and its possible evolutionary origin. Physiol. Plant. 100, 583–592. 10.1111/j.1399-3054.1997.tb03064.x

[B38] Johnson P. R. Ecker J. R. (1998). The ethylene gas signal transduction pathway: a molecular perspective. Annu. Rev. Genet. 32, 227–254. 10.1146/annurev.genet.32.1.227 9928480

[B39] Kang S. M. Khan A. L. Waqas M. You Y. H. Kim J. H. Kim J. G. (2014). Plant growth-promoting rhizobacteria reduce adverse effects of salinity and osmotic stress by regulating phytohormones and antioxidants in *Cucumis sativus*. J. Plant Interact. 9, 673–682. 10.1080/17429145.2014.894587

[B40] Khan A. L. Halo B. A. Elyassi A. Ali S. Al-Hosni K. Hussain J. (2016). Indole acetic acid and ACC deaminase from endophytic bacteria improves the growth of *Solanum lycopersicum*. Electron. J. Biotechnol. 21, 58–64. 10.1016/j.ejbt.2016.02.001

[B41] Kim H. E. Shitashiro M. Kuroda A. Takiguchi N. Kato J. (2007). Ethylene chemotaxis in *Pseudomonas aeruginosa* and other Pseudomonas species. Microb. Environ. 22, 186–189. 10.1264/jsme2.22.186

[B42] Klee H. J. Hayford M. B. Kretzmer K. A. Barry G. F. Kishore G. M. (1991). Control of ethylene synthesis by expression of a bacterial enzyme in transgenic tomato plants. Plant Cell. 3, 1187–1193. 10.1105/tpc.3.11.1187 1821764 PMC160085

[B43] Knief C. Delmotte N. Chaffron S. Stark M. Innerebner G. Wassmann R.. (2012). Metaproteogenomic analysis of microbial communities in the phyllosphere and rhizosphere of rice. ISME J. 6, 1378–1390. 10.1038/ismej.2011.192 22189496 PMC3379629

[B44] Kozdrój J. Van Elsas J. D. (2000). Response of the bacterial community to root exudates in soil polluted with heavy metals assessed by molecular and cultural approaches. Soil Biol. Biochem. 32, 1405–1417. 10.1016/S0038-0717(00)00058-4

[B45] Li J. Glick B. R. (2001). Transcriptional regulation of the *Enterobacter cloacae* UW4 1-aminocyclopropane-1-carboxylate (ACC) deaminase gene (acdS). Can. J. Microbiol. 47, 359–367. 10.1139/w01-009 11358176

[B46] Li J. McConkey B. J. Cheng Z. Guo S. Glick B. R. (2013). Identification of plant growth-promoting bacteria-responsive proteins in cucumber roots under hypoxic stress using a proteomic approach. J. Proteom. 84, 119–131. 10.1016/j.jprot.2013.03.011 23568019

[B47] Li J. Sun J. Yang Y. Guo S. Glick B. R. (2012). Identification of hypoxic-responsive proteins in cucumber using a proteomic approach. Plant Physiol. Biochem. 51, 74–80. 10.1016/j.plaphy.2011.10.011 22153242

[B48] Lieberman M. Kunishi A. Mapson L. W. Wardale D. (1966). Stimulation of ethylene production in apple tissue slices by methionine. Plant Physiol. 41, 376–382. 10.1104/pp.41.3.376 16656267 PMC1086352

[B49] Lorbiecke R. Sauter M. (1999). Adventitious root growth and cell-cycle induction in deepwater rice. Plant Physiol. 119, 21–30. 10.1104/pp.119.1.21 9880342 PMC32222

[B50] Martin M. N. Cohen J. D. Saftner R. A. (1995). A new 1-aminocyclopropane-1-carboxylic acid-conjugating activity in tomato fruit. Plant Physiol. 109, 917–926. 10.1104/pp.109.3.917 8552720 PMC161393

[B51] Mayak S. Tirosh T. Glick B. R. (2004). Plant growth-promoting bacteria confer resistance in tomato plants to salt stress. Plant Physiol. Biochem. 42, 565–572. 10.1016/j.plaphy.2004.05.009 15246071

[B52] Milly P. C. Wetherald R. T. Dunne K. A. Delworth T. L. (2002). Increasing risk of great floods in a changing climate. Nature 415, 514–517. 10.1038/415514a 11823857

[B53] Morgan P. W. Drew M. C. (1997). Ethylene and plant responses to stress. Physiol. Plant. 100, 620–630. 10.1111/j.1399-3054.1997.tb03068.x

[B54] Morris D. A. Larcombe N. J. (1995). Phloem transport and conjugation of foliar-applied 1-aminoc clopropane-1-carboxylic acid in cotton (*Gossypium hirsutum* L.). J. Plant Physiol. 146, 429–436. 10.1016/S0176-1617(11)82004-3

[B55] Najeeb U. Bange M. P. Tan D. K. Atwell B. J. (2015). Consequences of waterlogging in cotton and opportunities for mitigation of yield losses. AoB Plants 7:plv080. 10.1093/aobpla/plv080 26194168 PMC4565423

[B56] Nascimento F. Brígido C. Alho L. Glick B. R. Oliveira S. (2012). Enhanced chickpea growth-promotion ability of a Mesorhizobium strain expressing an exogenous ACC deaminase gene. Plant Soil 353, 221–230. 10.1007/s11104-011-1025-2

[B57] Nascimento F. X. Rossi M. J. Glick B. R. (2018). Ethylene and 1-Aminocyclopropane-1-carboxylate (ACC) in plant–bacterial interactions. Front. Plant Sci. 9:114. 10.3389/fpls.2018.00114 29520283 PMC5827301

[B58] Nascimento F. X. Rossi M. J. Soares C. R. McConkey B. J. Glick B. R. (2014). New insights into 1-aminocyclopropane-1-carboxylate (ACC) deaminase phylogeny, evolution and ecological significance. PLoS ONE 9:99168. 10.1371/journal.pone.0099168 24905353 PMC4048297

[B59] Nishiuchi S. Yamauchi T. Takahashi H. Kotula L. Nakazono M. (2012). Mechanisms for coping with submergence and waterlogging in rice. Rice 5:2. 10.1186/1939-8433-5-2 24764502 PMC3834488

[B60] Nonaka S. Yuhashi K. Takada K. Sugaware M. Minamisawa K. Ezura H. (2008). Ethylene production in plants during transformation suppresses vir gene expression in Agrobacterium tumefaciens. New Phytol. 178, 647–656. 10.1111/j.1469-8137.2008.02400.x 18331427

[B61] Osakabe Y. Osakabe K. Shinozaki K. Tran L. S. (2014). Response of plants to water stress. Front. Plant Sci. 5:86. 10.3389/fpls.2014.00086 24659993 PMC3952189

[B62] Paul M. V. Iyer S. Amerhauser C. Lehmann M. van Dongen J. T. Geigenberger P. (2016). Oxygen sensing via the ethylene response transcription factor RAP2. 12 affects plant metabolism and performance under both normoxia and hypoxia. Plant Physiol. 172, 141–153. 10.1104/pp.16.00460 27372243 PMC5074624

[B63] Pedersen O. Perata P. Voesenek L. A. C. J. (2017). Flooding and low oxygen responses in plants. Funct. Plant Biol. 44, III–VI. 10.1071/FPv44n9_FO 32480612

[B64] Peng H. P. Lin T. Y. Wang N. N. Shih M. C. (2005). Differential expression of genes encoding 1-aminocyclopropane-1-carboxylate synthase in Arabidopsis during hypoxia. Plant Mol. Biol. 58, 15–25. 10.1007/s11103-005-3573-4 16028113

[B65] Penrose D. M. Moffatt B. A. Glick B. R. (2001). Determination of 1-aminocycopropane-1-carboxylic acid (ACC) to assess the effects of ACC deaminase-containing bacteria on roots of canola seedlings. Can. J. Microbiol. 47, 77–80. 10.1139/w00-128 15049453

[B66] Rashid S. Charles T. C. Glick B. R. (2012). Isolation and characterization of new plant growth-promoting bacterial endophytes. Appl. Soil Ecol. 61, 217–224. 10.1016/j.apsoil.2011.09.011

[B67] Ravanbakhsh M. Sasidharan R. Voesenek L. A. Kowalchuk G. A. Jousset A. (2017). ACC deaminase-producing rhizosphere bacteria modulate plant responses to flooding. J. Ecol. 105, 979–986. 10.1111/1365-2745.12721

[B68] Reid M. S. (1995). Ethylene in plant growth, development, and senescence, in Plant Hormones, ed Davies P. J. (Dordrecht: Springer), 486–508.

[B69] Saleem M. Arshad M. Hussain S. Bhatti A. S. (2007). Perspective of plant growth promoting rhizobacteria (PGPR) containing ACC deaminase in stress agriculture. J. Indus. Microbiol. Biotechnol. 34, 635–648. 10.1007/s10295-007-0240-6 17665234

[B70] Sarkar A. Ghosh P. K. Pramanik K. Mitra S. Soren T. Pandey S. (2017). A halotolerant Enterobacter sp. displaying ACC deaminase activity promotes rice seedling growth under salt stress. Res. Microbiol. 4, 68–79. 10.1016/j.resmic.2017.08.005 28893659

[B71] Sasidharan R. Hartman S. Liu Z. Martopawiro S. Sajeev N. van Veen H.. (2017). Signal dynamics and interactions during flooding stress. Plant Physiol. 176, 1106–1117. 10.1104/pp.17.01232 29097391 PMC5813540

[B72] Sasidharan R. Voesenek L. A. (2015). Ethylene-mediated acclimations to flooding stress. Plant Physiol. 169, 3–12. 10.1104/pp.15.00387 25897003 PMC4577390

[B73] Setter T. L. Waters I. (2003). Review of prospects for germplasm improvement for waterlogging tolerance in wheat, barley and oats. Plant Soil 253, 1–34. 10.1023/A:1024573305997

[B74] Shiono K. Takahashi H. Colmer T. D. Nakazono M. (2008). Role of ethylene in acclimations to promote oxygen transport in roots of plants in waterlogged soils. Plant Sci. 175, 52–58. 10.1016/j.plantsci.2008.03.002

[B75] Singh R. P. Shelke G. M. Kumar A. Jha P. N. (2015). Biochemistry and genetics of ACC deaminase: a weapon to “stress ethylene” produced in plants. Front. Microbiol. 6:937 10.3389/fmicb.2015.00937 26441873 PMC4563596

[B76] Staswick P. E. Tiryaki I. (2004). The oxylipin signal jasmonic acid is activated by an enzyme that conjugates it to isoleucine in Arabidopsis. Plant Cell 16, 2117–2127. 10.1105/tpc.104.023549 15258265 PMC519202

[B77] Timmusk S. Paalme V. Pavlicek T. Bergquist J. Vangala A. Danilas T.. (2011). Bacterial distribution in the rhizosphere of wild barley under contrasting microclimates. PLoS ONE 6:e17968. 10.1371/journal.pone.0017968 21448272 PMC3063161

[B78] Vanderstraeten L. Van Der Straeten D. (2017). Accumulation and transport of 1-aminocyclopropane-1-carboxylic acid (ACC) in plants: current status, considerations for future research and agronomic applications. Front. Plant Sci. 8:38. 10.3389/fpls.2017.00038 28174583 PMC5258695

[B79] Vidoz M. L. Loreti E. Mensuali A. Alpi A. Perata P. (2010). Hormonal interplay during adventitious root formation in flooded tomato plants. Plant J. 63, 551–562. 10.1111/j.1365-313X.2010.04262.x 20497380

[B80] Viterbo A. Landau U. Kim S. Chernin L. Chet I. (2010). Characterization of ACC deaminase from the biocontrol and plant growth-promoting agent *Trichoderma asperellum* T203. FEMS Microbiol. Lett. 305, 42–48. 10.1111/j.1574-6968.2010.01910.x 20148973

[B81] Voesenek L. A. Sasidharan R. (2013). Ethylene–and oxygen signalling–drive plant survival during flooding. Plant Biol. 15, 426–435. 10.1111/plb.12014 23574304

[B82] Wang K. L. Li H. Ecker J. R. (2002). Ethylene biosynthesis and signaling networks. Plant Cell 14, S131–S151. 10.1105/tpc.001768 12045274 PMC151252

[B83] Wessjohann L. A. Brandt W. Thiemann T. (2003). Biosynthesis and metabolism of cyclopropane rings in natural compounds. Chem. Rev. 103, 1625–1648. 10.1021/cr0100188 12683792

[B84] Wright A. J. Kroon H. Visser E. J. Buchmann T. Ebeling A. Eisenhauer N.. (2017). Plants are less negatively affected by flooding when growing in species-rich plant communities. New Phytol. 213, 645–656. 10.1111/nph.14185 27717024

[B85] Xu M. Sheng J. Chen L. Men Y. Gan L. Guo S.. (2014). Bacterial community compositions of tomato (*Lycopersicum esculentum* Mill.) seeds and plant growth promoting activity of ACC deaminase producing Bacillus subtilis (HYT-12-1) on tomato seedlings. World J. Microbiol. Biotechnol. 30, 835–845. 10.1007/s11274-013-1486-y 24114316

[B86] Yang S. F. Hoffman N. E. (1984). Ethylene biosynthesis and its regulation in higher plants. Annu. Rev. Plant Physiol. 35, 155–189. 10.1146/annurev.pp.35.060184.001103

[B87] Yao M. Ose T. Sugimoto H. Horiuchi A. Nakagawa A. Wakatsuki S.. (2000). Crystal structure of 1-aminocyclopropane-1-carboxylate deaminase from *Hansenula saturnus*. J. Biol. Chem. 275, 34557–34565. 10.1074/jbc.M004681200 10938279

[B88] Zeng F. Shabala L. Zhou M. Zhang G. Shabala S. (2013). Barley responses to combined waterlogging and salinity stress: separating effects of oxygen deprivation and elemental toxicity. Front. Plant Sci. 4:313. 10.3389/fpls.2013.00313 23967003 PMC3743405

